# Improved Synthesis of *N*-Methylcadaverine

**DOI:** 10.3390/molecules23051216

**Published:** 2018-05-19

**Authors:** Kayla N. Anderson, Shiva Moaven, Daniel K. Unruh, Anthony F. Cozzolino, John C. D’Auria

**Affiliations:** Department of Chemistry and Biochemistry, Texas Tech University, Box 41061, Lubbock, TX 79409-1061, USA; kayla.anderson@ttu.edu (K.N.A.); shiva.moaven@ttu.edu (S.M.); daniel.unruh@ttu.edu (D.K.U.)

**Keywords:** alkaloid, granatane, *N*-methylcadaverine, *N*-methylpiperidine. reductive deamination

## Abstract

Alkaloids compose a large class of natural products, and mono-methylated polyamines are a common intermediate in their biosynthesis. In order to evaluate the role of selectively methylated natural products, synthetic strategies are needed to prepare them. Here, *N*-methylcadaverine is prepared in 37.3% yield in three steps. The alternative literature two-step strategy resulted in reductive deamination to give *N*-methylpiperidine as determined by the single crystal structure. A straightforward strategy to obtain the mono-alkylated aliphatic diamine, cadaverine, which avoids potential side-reactions, is demonstrated.

## 1. Introduction

Polyamines (PAs) are abundant in nature. Some of the most common examples include putrescine (1,4-diaminobutane), cadaverine (1,5-diaminopentane), spermidine (*N*-(3-aminopropyl)-1,4-diaminobutane), and spermine (*N*,*N*′-Bis(3-aminopropyl)-1,4-diaminobutane) [1]. Each of these PAs have been recruited by multiple lineages to perform various biological functions. For instance, all of the abovementioned PAs play a critical role as primary metabolites by mediating fundamental developmental processes [2]. More specifically, PAs in mammals and bacteria participate in the regulation of gene expression and gene transcription [1,3].

Plants utilize PAs for similar functions such as cell proliferation and cell signaling [2]. Additionally, PAs are employed for organ and pollen development [4]. PAs covalently bind to hydroxylcinnamate to form hyrdroxy-cinnamic acid amides (HCAAs), which drive pollen development and the pollen–pistil interaction during fertilization [4]. Contrary to mammals and bacteria, plants also utilize PAs for secondary metabolic purposes, such as stress responses [5].

Due to their sessile nature, plants produce phytoalexins and other specialized metabolites in order to mediate their responses with both the abiotic and biotic forces present in their surrounding environment [5]. Alkaloids comprise a large class of specialized metabolites that play key roles in these interactions. For example, steroidal alkaloids are known to cause inhibition of the fungal species *Phytophthora cactorum*, a known cause of root rot [6]. PAs have been implicated in alkaloid biosynthesis, specifically in piperidine and pyrrolidine alkaloids. Alkaloids are defined as nitrogen containing cyclic compounds. Alkaloids also have significant pharmacological properties. The alkaloids scopolamine and atropine are known for their anticholinergic and antispasmodic properties [7]. Alternatively, the alkaloids pseudopelleterine and *N*-methylpelleterine have historically been used for their anthelminthic (anti-worming) properties [5].

Scopolamine and atropine are compounds that originate from plants of the Solanaceae family. Both compounds belong to the class of alkaloids termed tropane alkaloids. Tropane alkaloids share a common *N*-methyl-8-azabicyclo[3.2.1]-octane core skeleton. Tropane alkaloids can also be categorized as a sub-class of pyrrolidine alkaloids because a pyrrolidine ring is part of the bicyclic structure. The PAs putrescine and spermidine are known intermediates of tropane alkaloid biosynthesis [7,8]. In addition, tropanes are constitutional isomers of granatane alkaloids, containing a one carbon difference in their bicyclic moieties.

**Figure 1:**
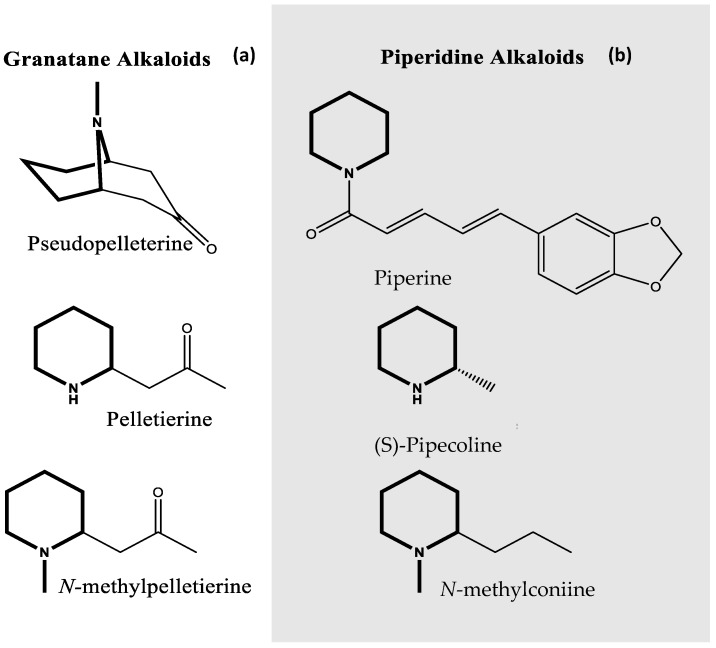
Natural products biosynthesized from *N*-methylcadaverine or *N*-methylpiperidine. The compounds in panel (**a**) are granatane alkaloids. The bolded atoms in panel (**a**) are from the proposed precursor *N*-methylcadaverine. The natural products in panel (**b**) are piperidine alkaloids. The bolded atoms show the piperidine ring from *N*-methylpiperidine.

**Figure 2:**
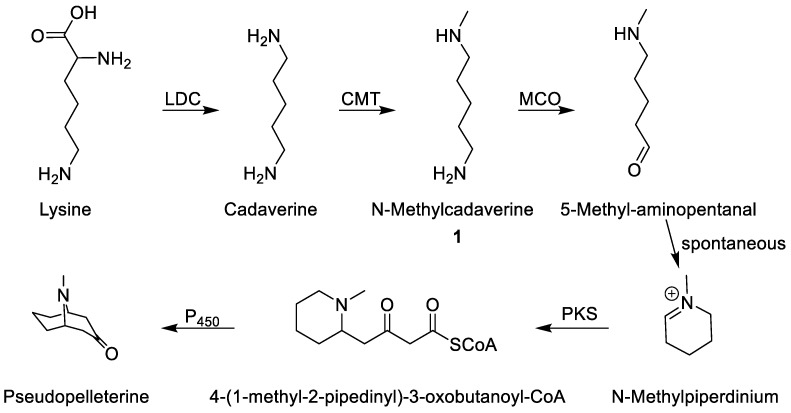
Proposed biosynthetic pathway of granatane alkaloid formation in *Punica grantum* [5]. The abbreviations above the arrows correspond with the presumed enzymes in the granatane pathway. LDC—lysine decarboxylase, CMT—cadaverine methyl-transferase, MCO—methylcadaverine oxidase, PKS—polyketide synthase P450—Cytochrome P450.

Granatane alkaloids are found predominantly in the species *P. granatum* (pomegranate). Granatane alkaloids are a sub-class of piperidine alkaloids, due to the presence of a piperidine ring in their core skeleton. The granatanes include the compounds *N*-methylpelleterine, pelleterine, and the bicyclic compound pseudopelletierine (*N*-methyl-9-azabicyclo[3.3.1]-nonane base structure) (Figure 1). Granatane alkaloids in *P. granatum* originate from the amino acid lysine. The evidence for this biosynthetic origin is based on the incorporation of radio-labeled [2-^14^C] lysine into the first ring of *N*-methylpelletierine during *in planta* feeding studies [9]. The results of these studies suggest a symmetrical intermediate in the formation of the piperidine ring. The symmetrical polyamine cadaverine is the product of the decarboxylation of lysine (Figure 2). When fed to whole pomegranate plants, radio-labeled [1,5-^14^C] cadaverine incorporated into the granatanes pelletierine, *N*-methylpelletierine, and pseudopelletierine. Additionally, the mono-methylation of cadaverine is a proposed enzymatic step in granatane alkaloid formation [5] (Figure 2). Currently, feeding studies using *N*-methylcadaverine (**1**) are not possible since commercial sources are not available. Therefore, a synthetic route to producing this compound would aid in the overall understanding of granatane biosynthesis.

Piperidine alkaloids compose a broader class of alkaloids also found in plants. Piperidine alkaloids are classified as compounds with a nitrogen containing six-membered core ring structure (Figure 1). However, piperidine alkaloids can be monocyclic or heterocyclic. Piperidine alkaloids are found in black pepper (*Psilocaulon absmile*) and poisonous hemlocks (*Conium maculatum*) (Figure 1) [10]. *N*-methylconiine, as well as other piperidine alkaloids found in *Conium maculatum* have been used for their analgesic abilities [11]. Substituted six-membered *N*-heterocycles are found in numerous natural products and pharmaceutical compounds that are commonly used today, such as the aforementioned *N*-methylconiine [12]. Hameed et al. (1992) utilized *N*-methylpiperidine (**2**) as a starting material for the synthesis of morphine analogs [13]. Alongside the synthesis of **2**, substituted *N*-heterocycles can be further synthesized for pharmaceutical purposes at a lesser cost [13].

A major hindrance to studying alkaloid biosynthesis is the lack of commercially available selectively *N*-methylated polyamines such as: **1**. To perform classical biochemical experiments on piperidine alkaloid producing plants, the synthesis of *N*-methylated polyamines is necessary. Monoalkylation of polyamines can present a challenge in achieving selectivity and also in limiting the extent of methylation [14,15]. Here, the synthesis of *N*-methylcadaverine and *N*-methylpiperidine by reductive amination of a nitrile is reported [16,17]. 

## 2. Results and Discussion

### 2.1. Synthesis of N-Methylpiperidinium Chloride *(**2***·HCl*)*

**Scheme 1:**
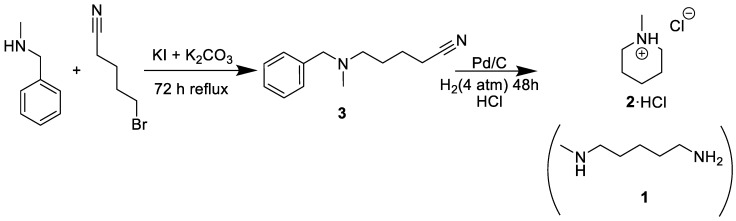
Synthesis of *N-*methylpiperidine hydrochloride (**2**·HCl) [1]. The proposed product **1**, is shown in parenthesis.

**Figure 3:**
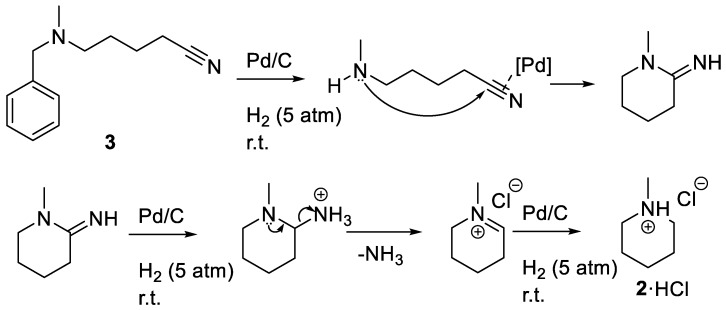
Proposed mechanism of cyclization from compound **3** to the final product **2**·HCl.

**Figure 4:**
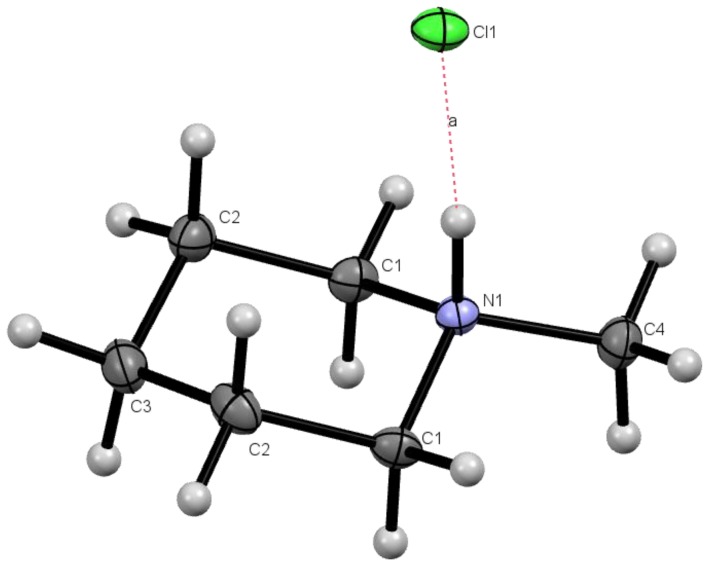
X-ray structure of **2**·HCl. The thermal ellipsoids are represented at 50% probability. Carbon, hydrogen, nitrogen, and chlorine atoms are represented by dark gray, light gray, purple, and green ellipsoids, respectively.

In an attempt to prepare *N*-methylcadaverine, the procedure reported by Jourdain, Caron, and Pommelet (Scheme 1) was followed [1]. *N*-Methylbenzylamine and 5-bromovaleronitrile react to yield 5-(benzyl(methyl)amino)pentanenitrile (**3**) (Scheme 1) as reported [1]. Both the reduction of the nitrile to a primary amine and the removal of the benzyl group were reported to proceed by hydrogenation over palladium on charcoal (Pd/C) (Scheme 1). Following the reported procedure, a material was recovered that did not match with **1** spectroscopically [1]. Instead, the ^1^H and ^13^C NMR spectra were consistent with a more symmetrical system with only four proton and carbon environments as opposed to the expected six (Figures S3 and S4). The mass spectrum suggested the loss of one amine. The product was concluded to be the cyclized product, *N*-methylpiperidinium chloride (**2**·HCl) (Figure S13). This species can be formed during the hydrogenation reaction, by initial deprotection of the tertiary amine to a secondary amine. This amine can attack the carbon of the Pd-activated nitrile to cyclize while forming the new C-N bond [16,17] (Figure 3). Reduction results first in deamination and finally the formation of **2**·HCl. The crystal structure of the observed product confirmed our conclusion that the synthesized product was **2**·HCl (Figure 4). In the structure, the chloride ion-pairs with the piperidinum through an NH hydrogen bond with a fairly typical Cl-N distance of 3.075 Å and is in close contact with CH hydrogen atoms on adjacent molecules [18,19]. All of the other metrical parameters are as expected.

### 2.2. Synthesis of N-Methylcadaverine Hydrochloride, *(**1**·2* HCl*)*

**Scheme 2:**
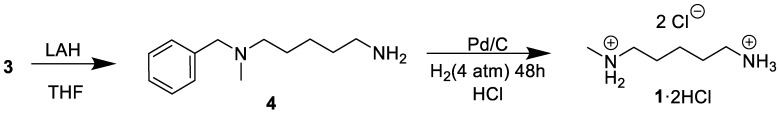
Synthesis of *N*-methylcadaverine hydrochloride (**1**·2HCl).

To obtain the desired *N*-methylcadaverine, the initial reaction scheme was revised (Scheme 2). The revised reaction still proceeds through **3**. To circumvent cyclization of the product, the nitrile was first reduced to a primary amine with lithium aluminum hydride (LAH). The reduction of the nitrile produced *N*^1^-benzyl-*N*^1^-methylpentane-1,5-diamine (4, Scheme 2). The removal of the benzyl group was subsequently achieved through a 48-h hydrogenation reaction over Pd/C. The desired product is isolated as the hydrochloride salt, **1**·2 HCl.

## 3. Materials and Methods

### 3.1. General Methods

The materials *N*-methybenzylamine (>97%, Tokyo Chemical Industry; Portaland, OR, USA), 5-bromovaleronitrile (95%, Santa Cruz Biotechnology; Dallas, TX, USA), Methanol (Fisher ACS grade; Madison, WI, USA), Ethanol (absolute, Pharmco; Toronto, ON, Canada), Potassium Iodide (99%, EMD Chemicals; Burlington, MA, USA), Potassium carbonate anhydrous (99%, EMD Chemicals; Burlington, MA, USA), Magnesium sulfate anhydrous (99%, J.T. Baker; Phillipsburg, NJ, USA), and Palladium/Carbon (10% wet, Oakwood Chemical; Estill, CA, USA) were used as purchased. Anhydrous tetrahydrofuran was obtained by passing HPLC grade THF over a bed of activated molecular sieves in a commercial (LC Technologies Solutions Inc.; Salisbury, MD, USA) solvent purification system (SPS). All NMR spectra were collected using a JEOL ECS 400 MHz NMR spectrometer (JEOL; Tokyo, Japan). All IR spectra were obtained using a Nicolet iS 5 FT-IR spectrometer equipped with a Specac Di Quest ATR accessory (Thermo Scientific; Madsion, WI, USA), high-resolution mass spectra (HRMS) were obtained on a Thermo Exactive MS with an Orbitrap mass analyzer in ESI mode, and CHN analysis were obtained on-site with a Perkin Elmer 2400 Series II CHNS/O Analyzer (Perkin Elmer; Waltham, MA, USA).

### 3.2. Synthesis of 5-(Benzyl(methyl)amino)pentanenitrile *(**3**)*

*N*-methylbenzylamine (121.18 g·mol^−1^, 6.05 g, 49.9 mmol) was dissolved in 150 mL of anhydrous ethanol and 10.32 g of potassium carbonate (138.20 g·mol^−1^, 74.67 mmol) and 1.24 g of potassium iodide (166.00 g·mol^−1^, 7.47 mmol) were suspended in the solution. The mixture was brought to reflux and 12.15 g of 5-bromopentanitrile (162.03 g·mol^−1^, 74.98 mmol) dissolved in 50 mL of anhydrous ethanol was added to the suspension dropwise over the course of 3 h. The solution was stirred under reflux for 72 h. Upon cooling, the salts were filtered off and the filtrate was taken to dryness. To the residue was added 100 mL of 2 M HCl solution and unreacted reagents were extracted via ether (3 × 50 mL). The aqueous layer was neutralized with 2 M NaOH solution and the final product was extracted with diethyl ether (3 × 100 mL). The organic solution was dried over MgSO_4_ and the solvent was removed. A light-yellow liquid (8.45 g) was collected giving **3** in 83.7% yield. **^1^H-NMR** (CDCl_3_) = 1.67 (m, 4H); 2.20 (s, 3H); 2.30 (t, 2H) (t, J = 6.6Hz); 2.38 (t, 2H) (t, J = 6.9Hz); 3.48 (s, 2H); 7.24–7.36 (m, 5H). **^13^C-NMR** (CDCl_3_) = 139.21 (s, 1C), 129.04 (s, 2C), 128.35 (s, 2C), 127.11 (s, 1C), 127.11 (s, 1C), 119.92 (s, 1C), 62.59 (s, 1C), 55.90 (s, 1C), 42.26 (s, 1C), 26.27 (s, 1C), 23.24 (s, 1C), 16.99 (s, 1C). **FTIR (ATR, cm^−1^)**: 3029 (s, C$sp^{2}$–H), 2949 (vs, C$sp^{3}$–H) 2245 (vs, C≡N).

### 3.3. Synthesis of N-Methylpiperidine Chloride *(**2***·HCl*)*

Compound **3** (203.17 g·mol^−1^, 3.95 g, 19.4 mmol) was dissolved in 15 mL of methanol and 1 mL of concentrated hydrochloric acid was added to the solution. The solution was transferred to a 250 mL Fisher-Porter bottle and 3.85 g of 10% (*w*/*w*) palladium on wet carbon was added to the reactor. The reactor was sealed and charged with 5 atm of H_2_. The mixture was stirred at room temperature for 24 h. The mixture was filtered through a bed of Celite to remove the Pd on carbon (Note that the Pd on carbon is pyrophoric at this stage and should not be allowed to dry or be placed in contact with organics). To crystalize the product as the hydrochloride salt, 1 mL of concentrated HCl was added to the solution. The volatile solvents were evaporated using a rotary-evaporator and the residual water was removed under high vacuum to give milky-white crystals (2.62 g, 93.4%). X-ray diffraction quality crystals of **2**·HCl were grown by slow cooling of supersaturated solution of **2**·HCl dissolved in hot acetone. **^1^H-NMR** (D_2_O spiked with acetone-*d*_5_) = 3.31, 3.27 (d, 2H), 2.76 (t, 2H), 2.65 (s, 3H), 1.78, 1.75 (d, 2H), 1.55 (m, 3H), 1.28 (m, 1H). **^13^C-NMR** (D_2_O spiked with Acetone-*d*_5_) = 20.62 (s, 1C), 22.99 (s, 2C), 43.16 (s, 1C), 54.91 (s, 1C). **FTIR (ATR, cm^−1^)**: 3005 (m, N^+^–H), 2947 (s, C$sp^{3}$–H). **HRMS** (ESI) *m/z*: [M + H]^+^ Calculated for C_6_H_14_N 100.1821; Found 100.1122.

### 3.4. Synthesis of N^1^-Benzyl-N^1^-methylpentane-1,5-diamine *(**4**)*

In a 250 mL round bottom flask, 100 mL of degassed anhydrous THF was added under a nitrogen atmosphere and 4.04 g of (**3**) (202.30 g·mol^−1^, 20.0 mmol) was added with a syringe through a septum. The solution was cooled in an ice bath and 4.54 g of lithium aluminum hydride (37.95 g·mol^−1^, 119 mmol) was added to the solution under a positive flow of nitrogen. After addition of lithium aluminum hydride, the mixture was allowed to warm to room temperature and subsequently refluxed for 48 h. After 48 h, the mixture was cooled to 0 °C and quenched using Fieser’s method [20]. The formed salts were removed by filtration and the remaining solution was taken to dryness to give the desired crude product as a light-yellow liquid. The crude product was further purified by column chromatography (first washed by DCM and next by MeOH–MeCN–Et_3_N (4:5:1)) (3.16 g, 76.6%) was collected. **^1^H**-**NMR** (CDCl_3_) = 7.29 (m. 5H), 3.45 (s, 2H), 2.66 (t, 2H), 2.34 (t, 2H), 2.15 (s, 3H), 1.50 (m, 2H), 1.42 (m, 2H), 1.33 (m, 2H). **^13^C**-**NMR** (CDCl_3_) = 139.30 (s, 1C), 129.15 (s, 2C), 128.26 (s, 2C), 126.96 (s, 1C), 62.45 (s, 1C), 57.51 (s, 1C), 42.35 (s, 1C), 42.24 (s, 1C), 33.74 (s, 1C), 27.35 (s, 1C), 24.77 (s, 1C). **FTIR (ATR, cm^−1^)**: 3357 (w, N–H), 3292 (w, N–H), 3025 (s, C$sp^{2}$–H), 2930 (s, C$sp^{3}$–H).

### 3.5. Synthesis of N-Methylcadaverine *(**1**·2* HCl*)*

A methanol (15 mL) solution of (**4**) (206.18 g·mol^−1^, 1 g, 4.8 mmol) was added to 250 mL Fisher-Porter bottle. Concentrated hydrochloric acid (0.25 mL) and 300 mg of 10% (*w*/*w*) palladium on wet carbon were added to the reactor respectively and it was charged with hydrogen gas (60 psi). The mixture was stirred at room temperature for 48 h. The mixture was filtered, and 1 mL of concentrated hydrochloric acid solution was added to this solution and then it was taken to dryness to give *N*-methyl cadaverine as a white solid. (549 mg, 60.2%. **^1^H**-**NMR** (D_2_O) = 2.79(m, 4H), 2.49 (s, 3H), 1.51 (m, 4H), 1.26 (m. 2H). **^13^C**-**NMR** (Acetonitrile-*d*_6_) = 48.93 (s, 1C), 39.36 (s, 1C), 32.86 (s, 1C), 27.21 (s, 1C), 25.31 (s, 1C), 22.84 (s, 1C). **FTIR (ATR, cm^−1^)**: 3011(vw, N^+^–H), 2729 (s, N^+^–H), 2932 (s, C$sp^{3}$–H). **HRMS** (ESI) *m/z*: [M + H]^+^ Calculated for C_6_H_17_N_2_ 117.2126; found 117.1386.

## 4. Conclusions

A synthetic method for mono-methylated polyamines is necessary to continue biochemical analysis of piperidine alkaloid biosynthesis. The three-step method used in this paper allows for the straightforward synthesis of the mono-methylated polyamine, cadaverine, without a possible side reaction to *N*-methylpiperdine.

## Supplementary Materials

Images of all ^1^H-, ^13^C-NMR and FTIR spectra are available online. The crystal structure information for compound **2**·HCl was deposited with the CCDC as a private communication with deposition number 1542426.
