# Supplementary material for: Improved Synthesis of N-Methylcadaverine
Source: Molecules. 2018 May 19;23(5):1216. doi: 10.3390/molecules23051216 (PMC6100435; doi:10.3390/molecules23051216)
Supplement: Supplementary file 1 [file molecules-23-01216-s001.pdf]

Supplementary Information

Improved Synthesis of *N*-Methylcadaverine

Kayla N. Anderson, Shiva Moaven, Daniel K. Unruh, Anthony F. Cozzolino * and John C. D'Auria *

Department of Chemistry and Biochemistry, Texas Tech University, Box 41061, Lubbock, TX 79409-1061, USA;

Table of Contents

S1 Crystallographic Details 2

S2 Spectroscopic Data 6

S3 References 13

S1 Crystallographic Details

General Data Collection

Data were collected on a Bruker PLATFORM three circle diffractometer equipped with an APEX II CCD detector and operated at 1350 W (50kV, 30 mA) to generate (graphite-monochromated) Mo Kα radiation (λ = 0.71073 Å). Crystals were transferred from the vial and placed on a glass slide in polyisobutylene. A Zeiss Stemi 305 microscope was used to identify a suitable specimen for X-ray diffraction from a representative sample of the material. The crystal and a small amount of the oil were collected on a MĭTiGen cryoloop and transferred to the instrument, where it was placed under a cold nitrogen stream (Oxford) maintained at 100 K throughout the duration of the experiment. The sample was optically centered with the aid of a video camera to ensure that no translations were observed as the crystal was rotated through all positions.

A unit cell collection was then carried out. After it was determined that the unit cell was not present in the CCDC database a sphere of data was collected. Omega scans were carried out with a 10 sec/frame exposure time and a rotation of 0.50° per frame. After data collection, the crystal was measured for size, morphology, and color. These values are reported in Table S1.

Refinement Details

After data collection, the unit cell was re-determined using a subset of the full data collection. Intensity data were corrected for Lorentz, polarization, and

background effects using the Bruker program APEX 3 [1]. A semi-empirical correction for adsorption was applied using the program *SADABS* [2]. The *SHELXL-2014* series of programs was used for the solution and refinement of the crystal structure [3]. Hydrogen atoms bound to carbon and nitrogen atoms were located in the difference Fourier map and were geometrically constrained using the appropriate AFIX commands. The hydrogen atoms bound to C4 could not reach a stable configuration using an AFIX 137 command, so the AFIX 33 constraint was used with a PART-1 command to help delineate symmetrically equivalent atoms. The RIGU restraint was also applied globally during the final refinements.

Table S1. Crystal data and structure refinement for **2**·HCl.

Crystal Color	colorless
Crystal Habit	blocky
Empirical formula	$C_6H_{14}ClN$
Formula weight	135.13
Temperature	100(2) K
Wavelength	0.71073 A
Crystal system	Orthorhombic
Space group	*Pnma*
Unit cell dimensions	a = 15.874(2) Å alpha = 90 °.
	b = 6.8527(9) Å beta = 90 °.
	c = 7.0867(9) Å gamma = 90 °.
Volume	770.88(17) Å^3
Z	4
Calculated density	1.164 mg/m^3
Absorption coefficient	0.402 mm^{-1}
F(000)	294
Crystal size	0.325 x 0.305 x 0.175 mm
Theta range for data collection	2.566 to 27.128 °.
Limiting indices	$-20\le h\le 20$, $-8\le k\le 8$, $-9\le l\le 9$
Reflections collected / unique	8323 / 928 [R(int) = 0.0221]

Completeness to theta = 25.242°	100.0 %
Refinement method	Full-matrix least-squares on F^2
Data / restraints / parameters	928 / 21 / 43
Goodness-of-fit on F^2	1.081
Final R indices [I>2sigma(I)]	R1 = 0.0253, wR2 = 0.0636
R indices (all data)	R1 = 0.0274, wR2 = 0.0648
Largest diff. peak and hole	0.316 and -0.259 e.A^{-3}

Table S2. Bond lengths [Å] and angles [°] for **2**·HCl.

N(1)-C(4)	1.488(2)
N(1)-C(1)#1	1.4981(13)
N(1)-C(1)	1.4981(13)
N(1)-H(1)	1.0000
C(1)-C(2)	1.5218(16)
C(1)-H(1A)	0.9900
C(1)-H(1B)	0.9900
C(2)-C(3)	1.5252(16)
C(2)-H(2A)	0.9900
C(2)-H(2B)	0.9900
C(3)-C(2)#1	1.5252(16)
C(3)-H(3A)	0.9900
C(3)-H(3B)	0.9900
C(4)-H(4A)	0.9800
C(4)-H(4B)	0.9800
C(4)-H(4C)	0.9800
C(4)-N(1)-C(1)#1	111.60(8)
C(4)-N(1)-C(1)	111.59(8)
C(1)#1-N(1)-C(1)	111.13(12)
C(4)-N(1)-H(1)	107.4
C(1)#1-N(1)-H(1)	107.4
C(1)-N(1)-H(1)	107.4
N(1)-C(1)-C(2)	109.87(10)
N(1)-C(1)-H(1A)	109.7
C(2)-C(1)-H(1A)	109.7
N(1)-C(1)-H(1B)	109.7
C(2)-C(1)-H(1B)	109.7
H(1A)-C(1)-H(1B)	108.2
C(1)-C(2)-C(3)	111.49(11)
C(1)-C(2)-H(2A)	109.3
C(3)-C(2)-H(2A)	109.3
C(1)-C(2)-H(2B)	109.3
C(3)-C(2)-H(2B)	109.3
H(2A)-C(2)-H(2B)	108.0
C(2)-C(3)-C(2)#1	110.87(13)
C(2)-C(3)-H(3A)	109.5
C(2)#1-C(3)-H(3A)	109.5
C(2)-C(3)-H(3B)	109.5
C(2)#1-C(3)-H(3B)	109.5
H(3A)-C(3)-H(3B)	108.1
N(1)-C(4)-H(4A)	109.5
N(1)-C(4)-H(4B)	109.5
H(4A)-C(4)-H(4B)	109.5
N(1)-C(4)-H(4C)	109.5
H(4A)-C(4)-H(4C)	109.5
H(4B)-C(4)-H(4C)	109.5

Symmetry transformations used to generate equivalent atoms:
#1 x,-y+1/2,z

Table S3. Hydrogen bonds for **2**·HCl where hydrogen bonds with H..A < r(A) + 2.000 Å and <DHA > 110° are listed.

D-H	d(D-H)	d(H..A)	<DHA	d(D..A)	A
N1-H1	1.000	2.077	175.31	3.075	Cl1

S2 Spectroscopic Data

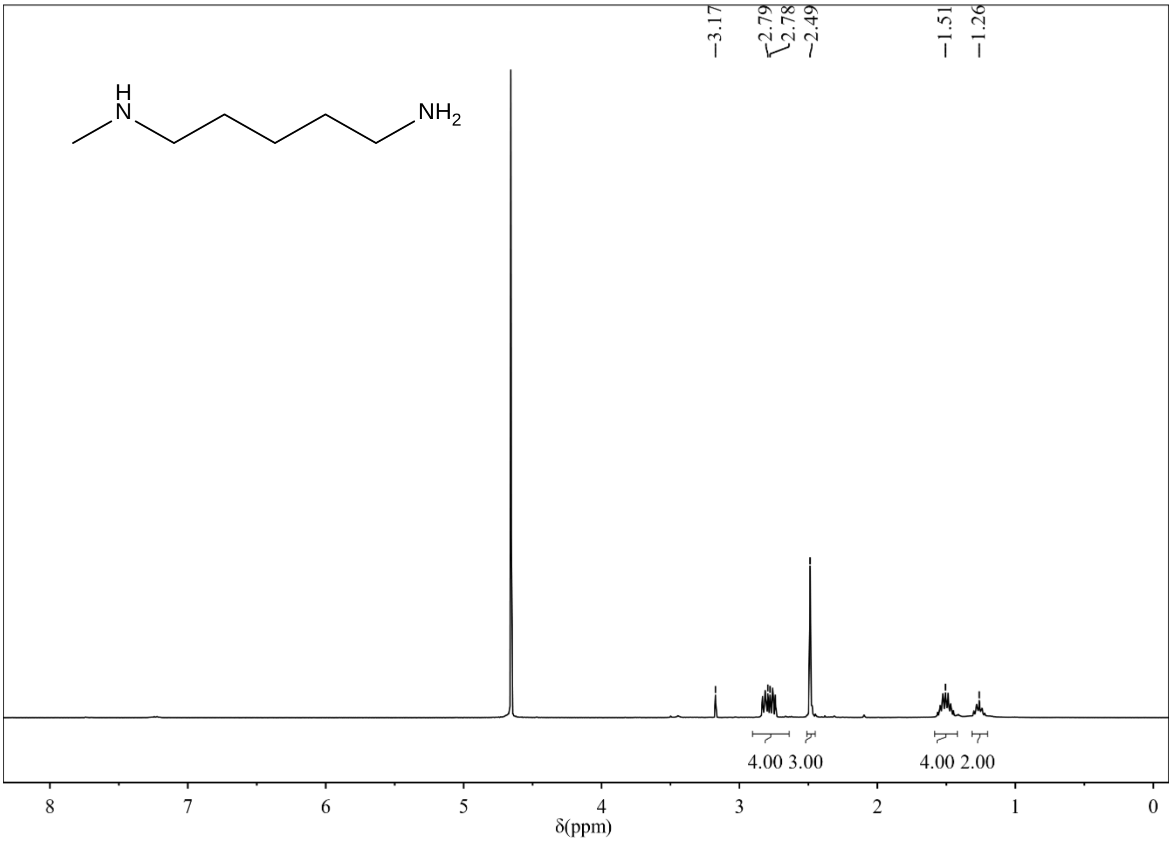

Figure S1. ^{1}H NMR spectra for compound **1**·2HCl

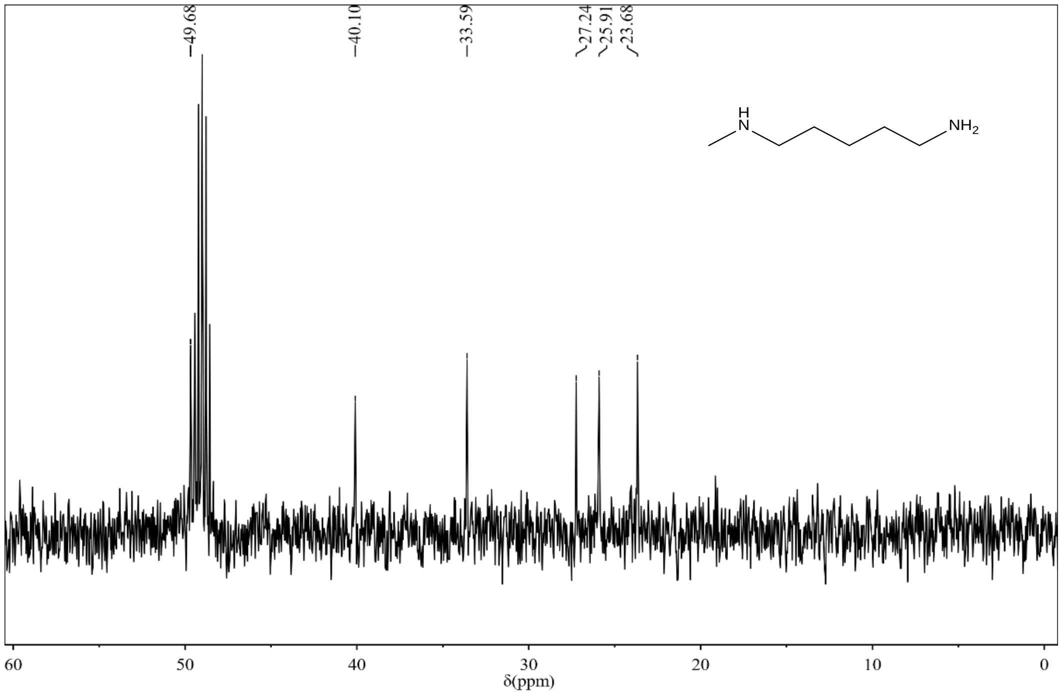

Figure S2. ^{13}C NMR of compound **1**·2HCl.

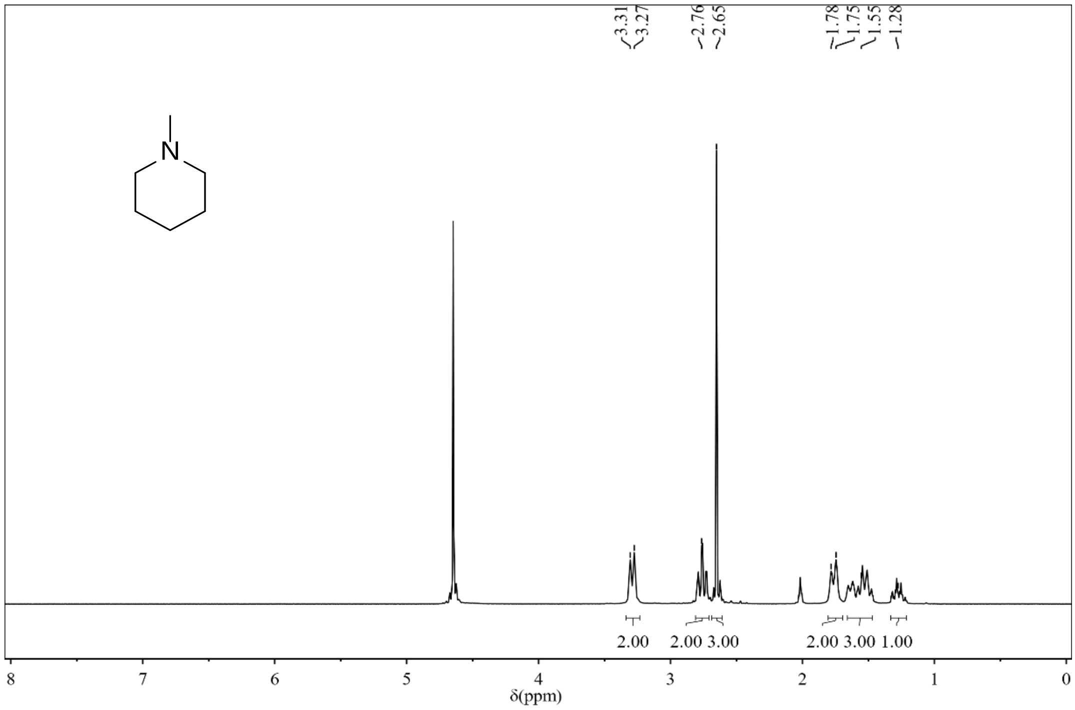

Figure S3. ^{1}H NMR spectra for compound **2**·HCl.

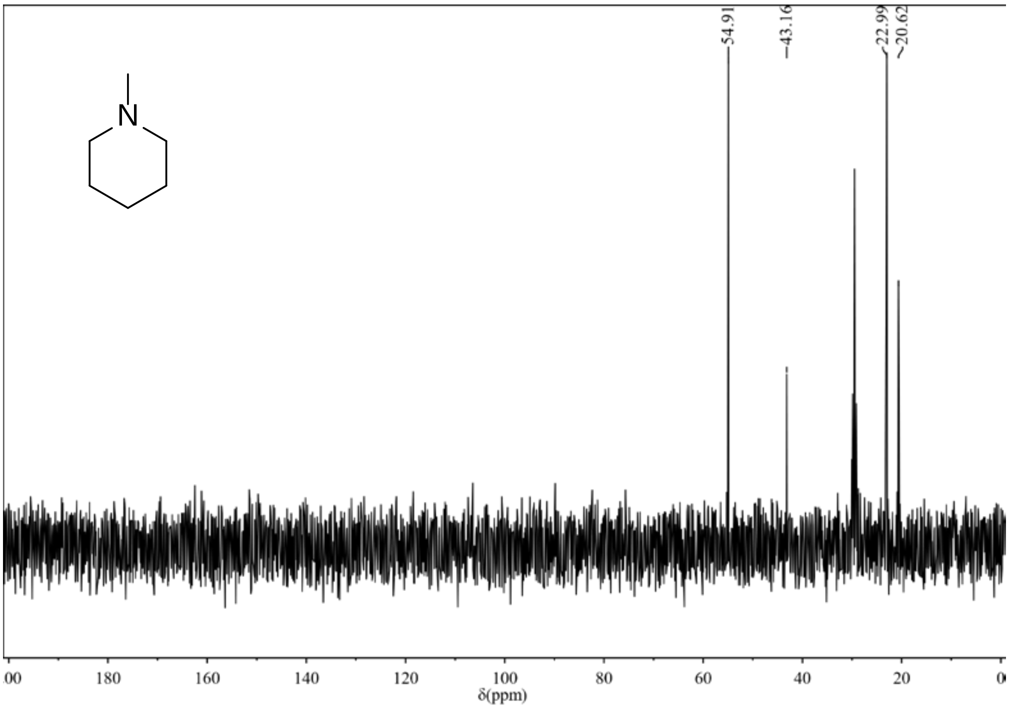

Figure S4. ^{13}C NMR of compound **2**·HCl.

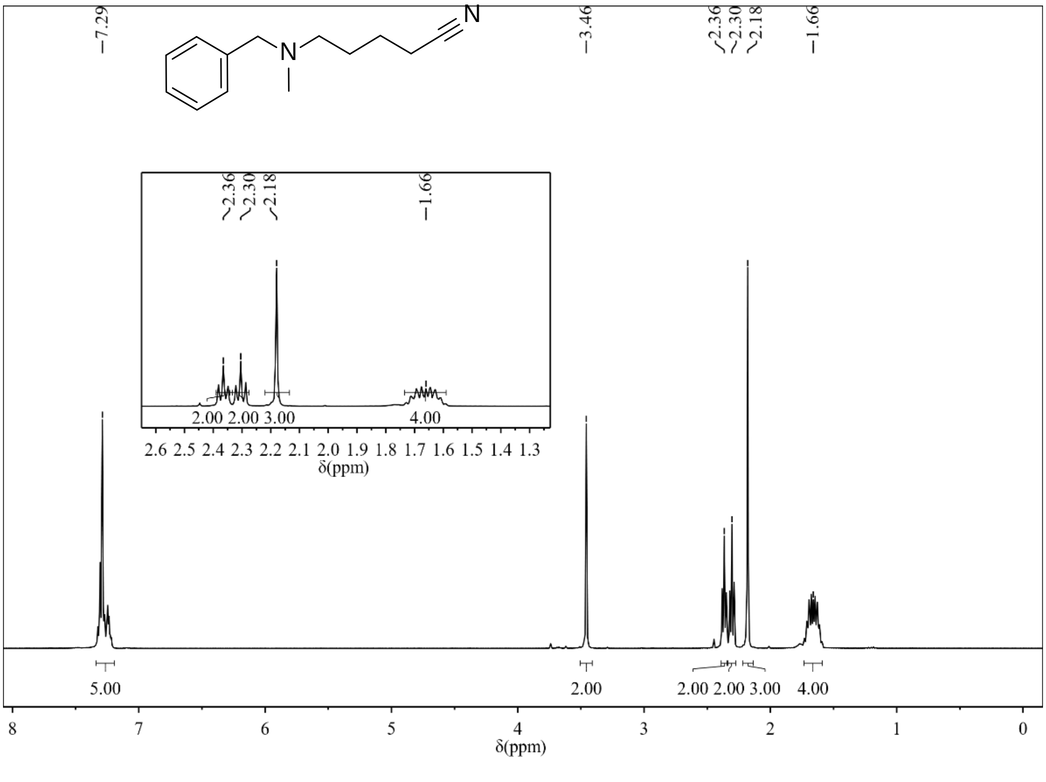

Figure S5. ^{1}H NMR of compound **3**

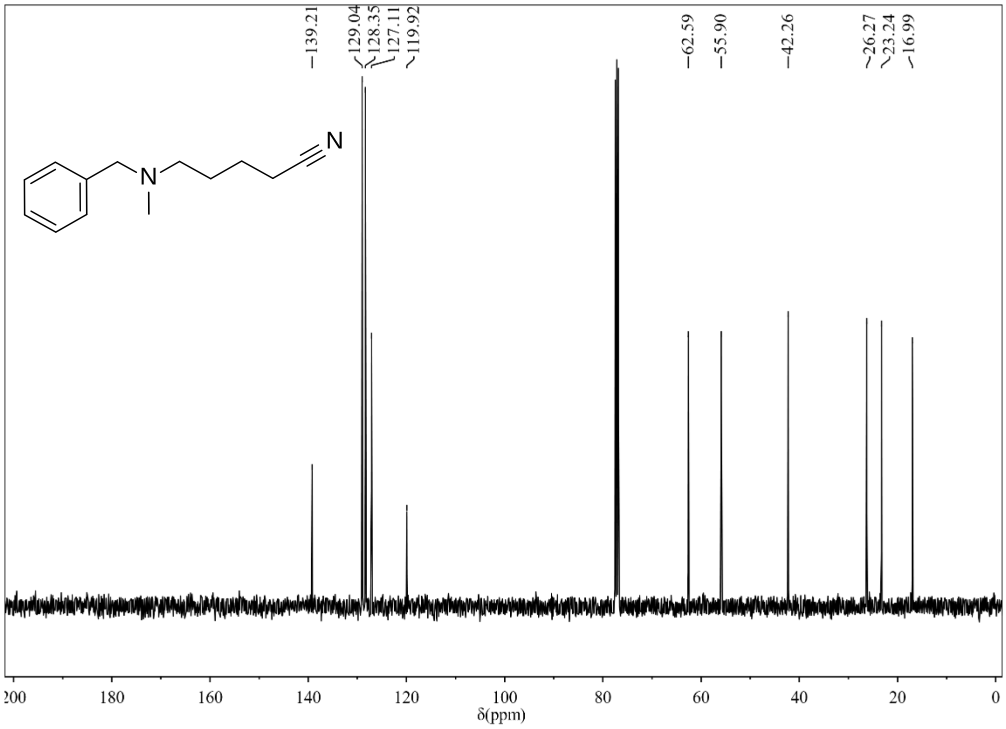

Figure S6. ^{13}C NMR spectra of compound **3**.

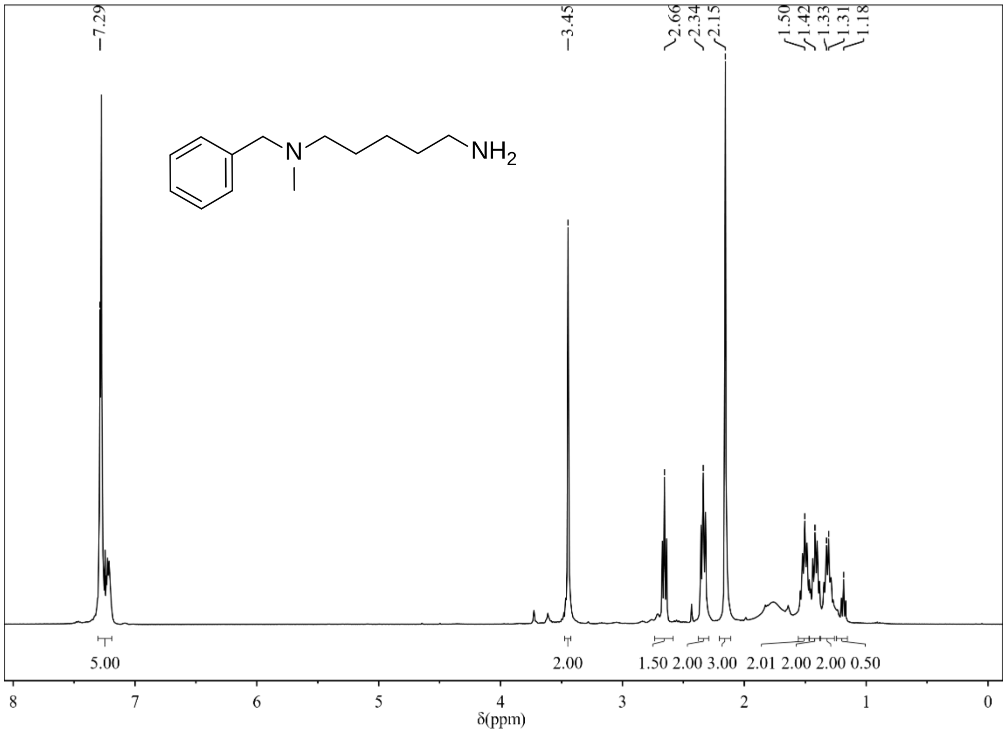

Figure S7. ^{1}H NMR of compound **4**.

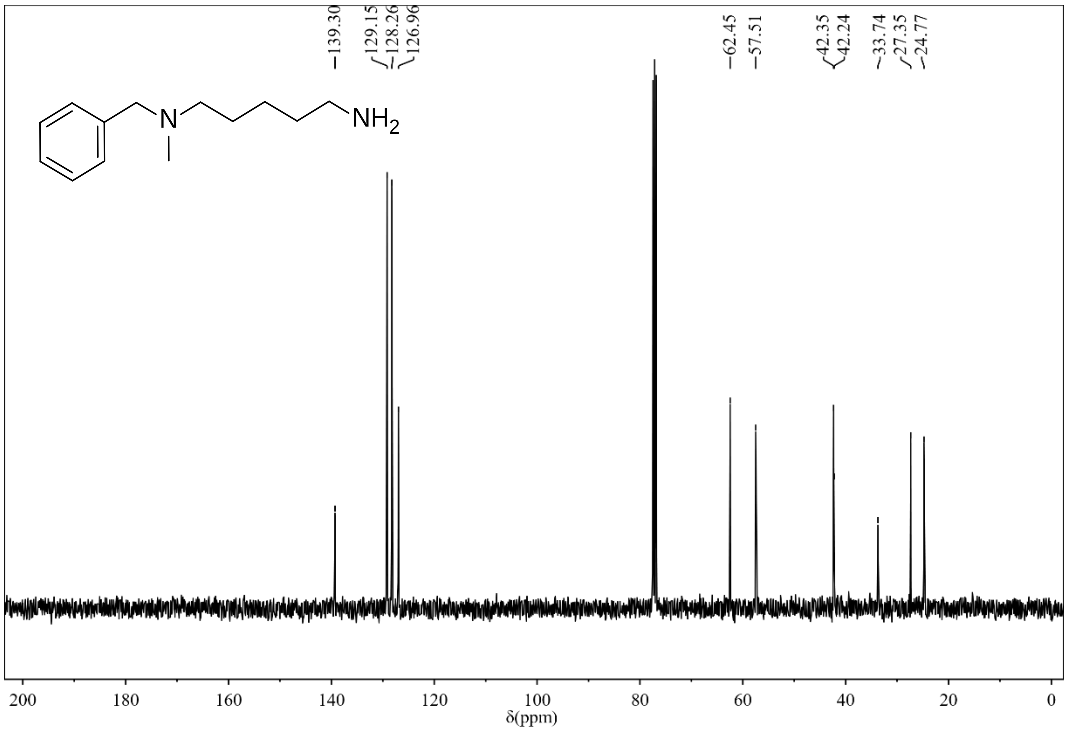

Figure S8. ^{13}C NMR of compound **4**.

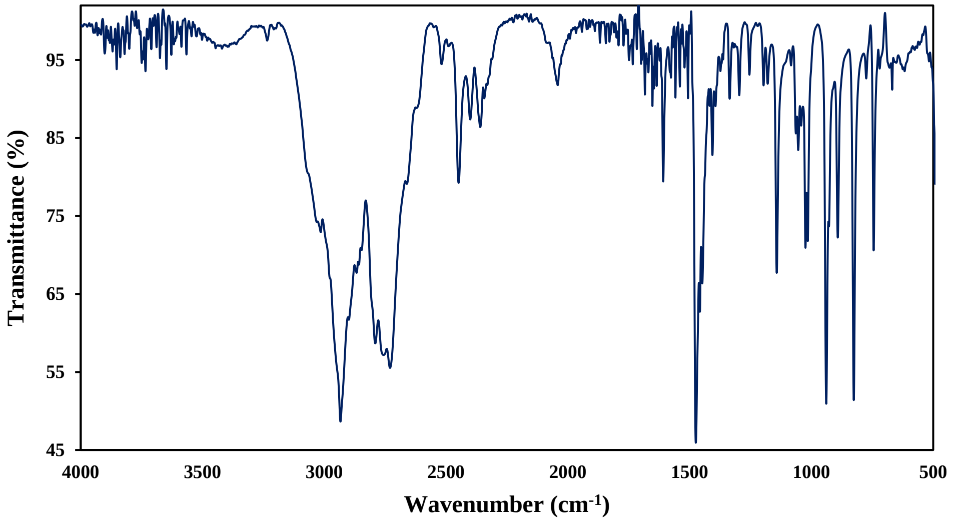

Figure S9. Di-ATR FTIR of compound **1**·2HCl

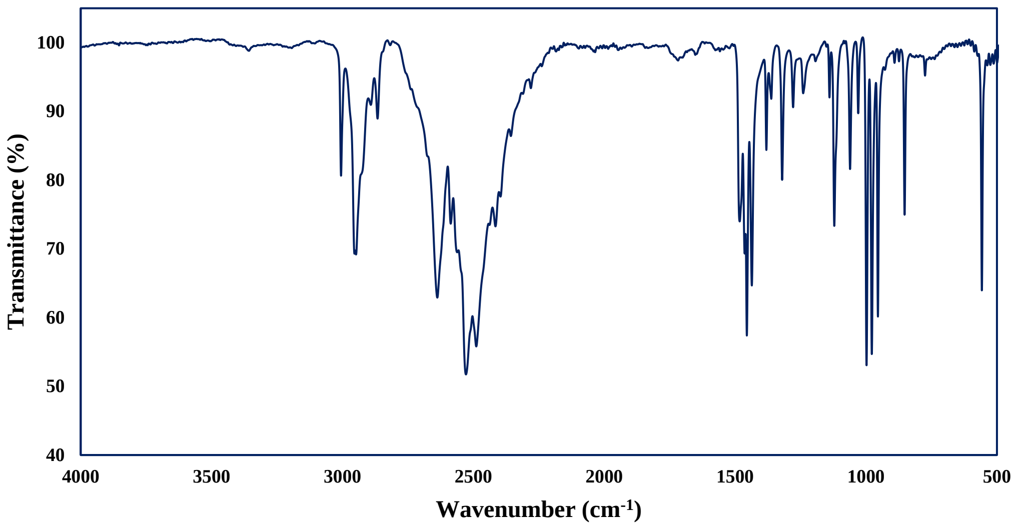

Figure S10. Di-ATR FTIR of compound **2**·HCl.

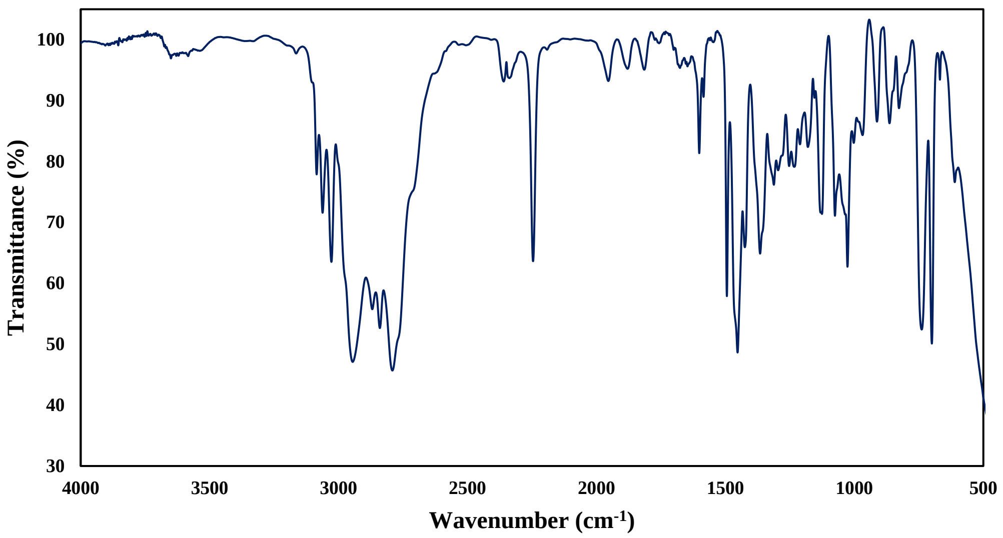

Figure S11. Di-ATR FTIR of compound **3**.

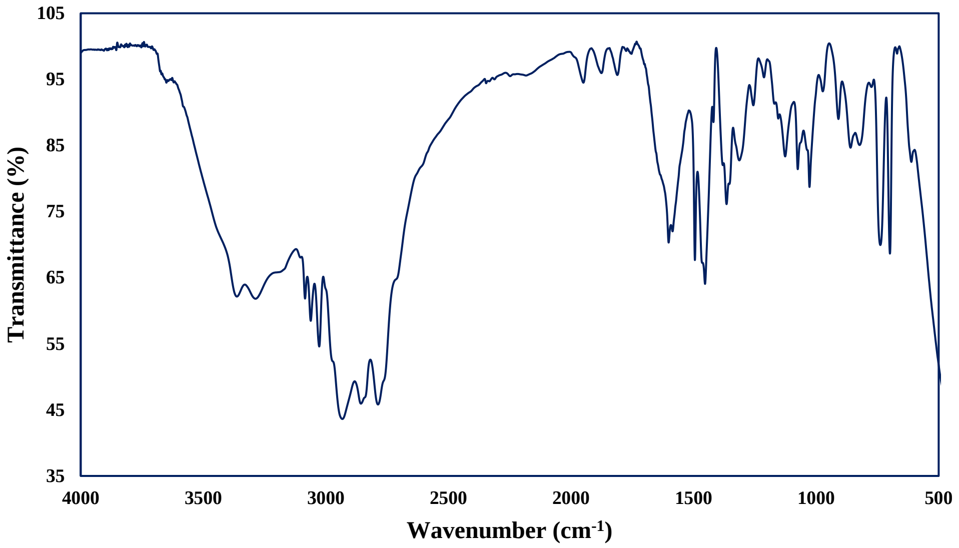

Figure S12. Di-ATR FTIR of compound **4**.

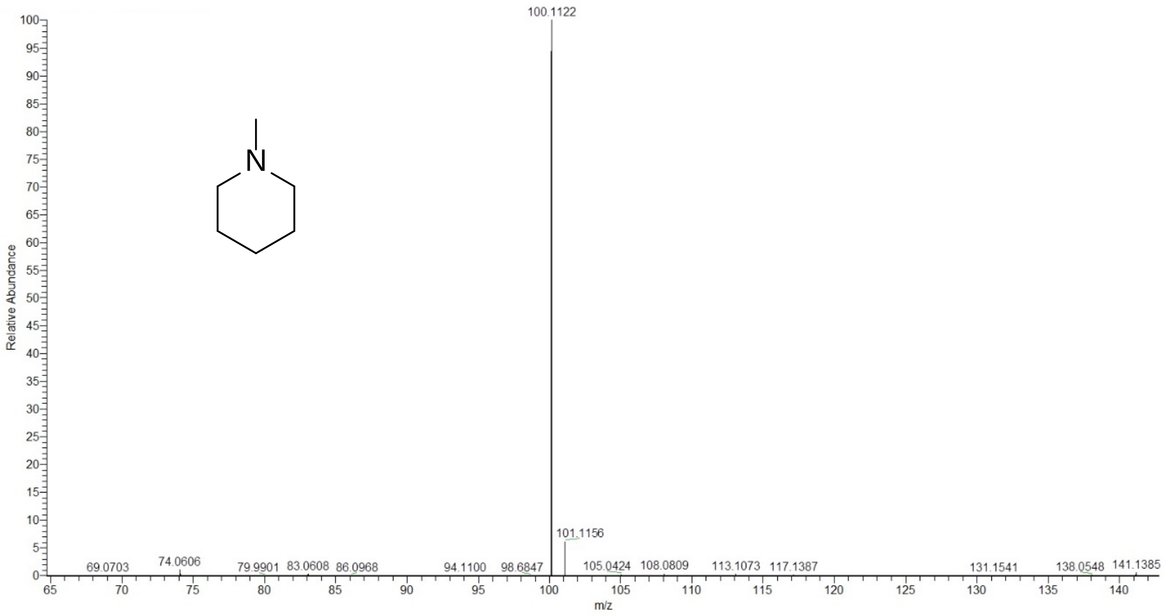

Figure S13. HR-MS in positive mode of compound **2**·HCl with ESI method, 1mg/mL sample in methanol. MW of *N*-methylpiperidine = 99.18 g/mole and m/z+1= 100.1.

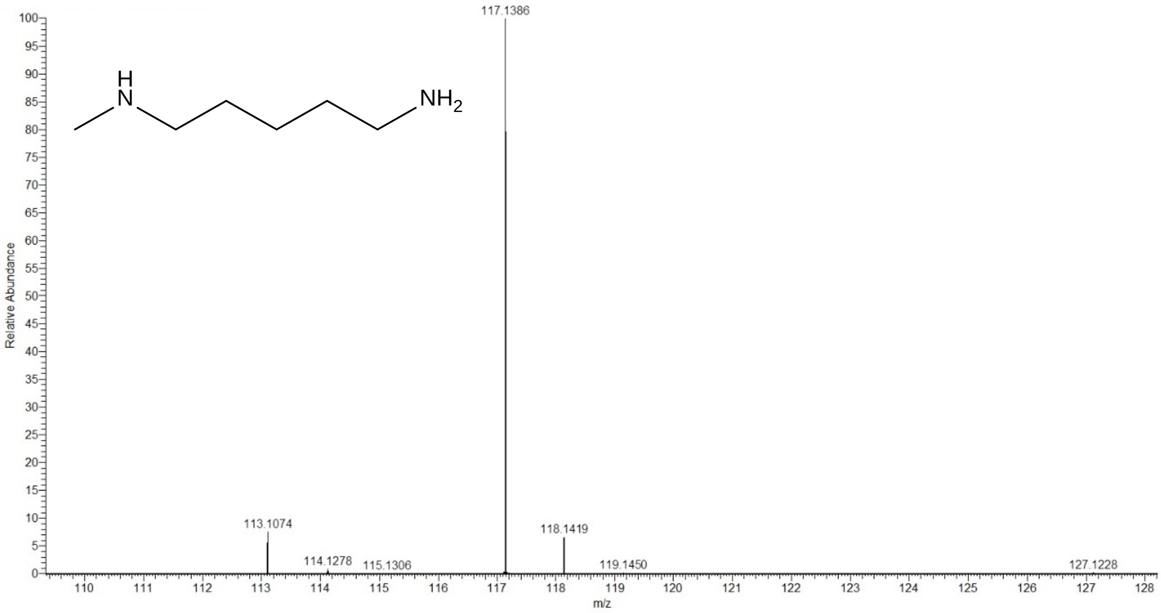

Figure S14. HR-MS in positive mode of compound **1**·2HCl with ESI method, 1mg/mL sample in methanol. MW of *N*-methylcadaverine = 116.13 g/mole and m/z+1= 117.13.

S3 References

1. APEX3 Data Collection Software, Version 2016.5-0; Bruker AXS: Delft, The Netherlands, 2016;
2. Sheldrick, G. i SADABS, program for empirical absorption correction of area detector data. Univ. Gött. Ger. **1996**.
3. Sheldrick, G. M. Crystal structure refinement with SHELXL. Acta Crystallogr. Sect. C Struct. Chem. **2015**, 71, 3–8, doi:10.1107/S2053229614024218.
